# Changes in Bleaching Susceptibility among Corals Subject to Ocean Warming and Recurrent Bleaching in Moorea, French Polynesia

**DOI:** 10.1371/journal.pone.0070443

**Published:** 2013-07-29

**Authors:** Morgan S. Pratchett, Dominique McCowan, Jeffrey A. Maynard, Scott F. Heron

**Affiliations:** 1 Australian Research Council Centre of Excellence for Coral Reef Studies, James Cook University of North Queensland, Townsville, Queensland, Australia; 2 Laboratoire d’Excellence (CORAIL) USR 3278 CNRS – EPHE, CRIOBE, Papetoai, Moorea, Polynésie Française; 3 Center for Marine Science, CREST Research Park of UNCW, Wilmington, North Carolina, United States of America; 4 National Oceanic & Atmospheric Administration Coral Reef Watch, Townsville, Queensland, Australia; 5 Marine Geophysical Laboratory, School of Engineering and Physical Sciences, James Cook University, Townsville, Queensland, Australia; Centrum Wiskunde & Informatica (CWI) & Netherlands Institute for Systems Biology, Netherlands

## Abstract

**Background:**

Climate-induced coral bleaching poses a major threat to coral reef ecosystems, mostly because of the sensitivities of key habitat-forming corals to increasing temperature. However, susceptibility to bleaching varies greatly among coral genera and there are likely to be major changes in the relative abundance of different corals, even if the wholesale loss of corals does not occur for several decades. Here we document variation in bleaching susceptibility among key genera of reef-building corals in Moorea, French Polynesia, and compare bleaching incidence during mass-bleaching events documented in 1991, 1994, 2002 and 2007.

**Methodology/Principal Findings:**

This study compared the proportion of colonies that bleached for four major genera of reef-building corals (*Acropora*, *Montipora*, *Pocillopora* and *Porites*), during each of four well-documented bleaching events from 1991 to 2007. *Acropora* and *Montipora* consistently bleached in far greater proportions (up to 98%) than *Pocillopora* and *Porites*. However, there was an apparent and sustained decline in the proportion of colonies that bleached during successive bleaching events, especially for *Acropora* and *Montipora*. In 2007, only 77% of *Acropora* colonies bleached compared with 98% in 1991. Temporal variation in the proportion of coral colonies bleached may be attributable to differences in environmental conditions among years. Alternately, the sustained declines in bleaching incidence among highly susceptible corals may be indicative of acclimation or adaptation.

**Conclusions/Significance:**

Coral genera that are highly susceptible to coral bleaching, and especially *Acropora* and *Montipora*, exhibit temporal declines in their susceptibility to thermal anomalies at Moorea, French Polynesia. One possible explanation for these findings is that gradual removal of highly susceptible genotypes (through selective mortality of individuals, populations, and/or species) is producing a coral assemblage that is more resistant to sustained and ongoing ocean warming.

## Introduction

Disturbances have an important influence on the structure and dynamics of shallow marine environments [1], and especially for coral reef ecosystems [2,3]. The frequency and severity of episodic disturbances has increased greatly in recent years due to emerging effects of global climate change combined with increasing anthropogenic pressures ( [1,4]). Increases in disturbance frequency, severity and diversity are leading to widespread degradation of coral reef ecosystems. Wilkinson [5] estimated that 20% of the world’s coral reefs have already been severely degraded, whereby coral cover has declined by >90% and there is limited prospect of recovery. Coral-reef degradation is mostly concentrated in eastern Africa, South-East Asia, and the central and southern Caribbean [5] in areas with large human populations and a long history of exploitation [6]. Coral reef degradation is therefore caused, or at least precipitated, by direct anthropogenic disturbances. Climate change is also contributing to recent coral reef degradation [5,7], and is expected to become the major cause of habitat degradation on reefs toward the latter part of this century [8].

The most apparent effects of climate change in natural ecosystems are changes in phenology (e.g., altered timing of reproductive activities [9,10]) and shifts in species ranges [11–13]. Climate change has also caused dramatic shifts in the relative abundance of species (e.g., arctic, arid, tropical rainforests and coral reef ecosystems), and has contributed to species extinctions [10,13]. Thomas et al. [13] predicted that 15–37% of species in terrestrial ecosystems will become extinct given a 2.0°C increase in mean global atmospheric temperatures. Significant species losses will not only reduce biological diversity but also potentially undermine ecosystem function, increasing the likelihood of ecosystem collapse [14,15].

On coral reefs, climate change (specifically, anomalously high sea temperatures) has been linked to large-scale coral bleaching [16–18]. Many reef-building corals function close to their upper thermal limits, such that small increases in maximum temperatures (as little as 1.0°C [18]) lead to a breakdown in the relationship between the coral host and the critical photosynthesizing symbiotic zooxanthellae that give corals their colour. Declines in the densities of zooxanthellae leave corals ‘bleached’. The increasing occurrence and severity of climate-induced coral bleaching reflects gradual increases in global sea-surface temperatures (SST). On average, global SST has increased by 0.7°C in the last century [8], bringing baseline ocean temperatures much closer to the maximum thermal tolerances for reef-building corals. As a consequence, naturally occurring temperature anomalies (e.g., linked to El Niño) increasingly cause thermal tolerances of corals to be exceeded, resulting in more frequent and severe episodes of coral bleaching [7,19].

Climate-change models predict a further 1.8–4°C increase in temperatures for tropical regions over the next century [8]. By 2050, most coral reefs are expected to be subject to annual thermal anomalies equivalent to the conditions in 1998 [8,20], which caused extensive coral bleaching in every ocean, and killed up to 90% of coral on individual reefs. Unless corals can adapt, ocean warming could cause wholesale loss of corals within the 21^st^ century [7], leading to fundamental shifts in the structure and function of coral reef ecosystems [21]. While it is likely that corals can adapt to changing environmental conditions, it is unclear whether adaptation can occur fast enough to ensure long-term persistence of coral-dominated ecosystems [22]). However, mass bleaching (especially when accompanied by highly selective mortality) is likely to impose strong selective pressures on coral populations and communities [23], leading to rapid increases in the prevalence of corals that are resistant or resilient to high temperatures [24,25].

If corals can adapt or acclimate to ocean warming, we would expect a temporal decline in the proportion of coral colonies that bleach, or die, at a given temperature. In Moorea, French Polynesia, in the central Pacific, major episodes of coral bleaching have occurred every 2–5 years since 1991 [26], corresponding with significant positive temperature anomalies [7,27]. This study quantified the proportion of coral colonies that bleached and/or died during a mass-bleaching event in late summer 2007. Our findings were then compared with well-documented and comparable bleaching events in 1991, 1994, and 2002, testing for declines in bleaching susceptibility among key genera of reef-building corals. There was evidence of temporal declines in bleaching susceptibility in some (but not all) coral genera, which is suggestive of population acclimation or community-level adaptation. While climate-change will undoubtedly cause, and is already having, major impacts on coral reef ecosystems [8,28], variation in the current and future capacity of corals to resist bleaching will lead to marked changes in community composition, prior to wholesale loss of these important habitat-forming organisms.

## Results

### Recent bleaching

**Figure 1:**
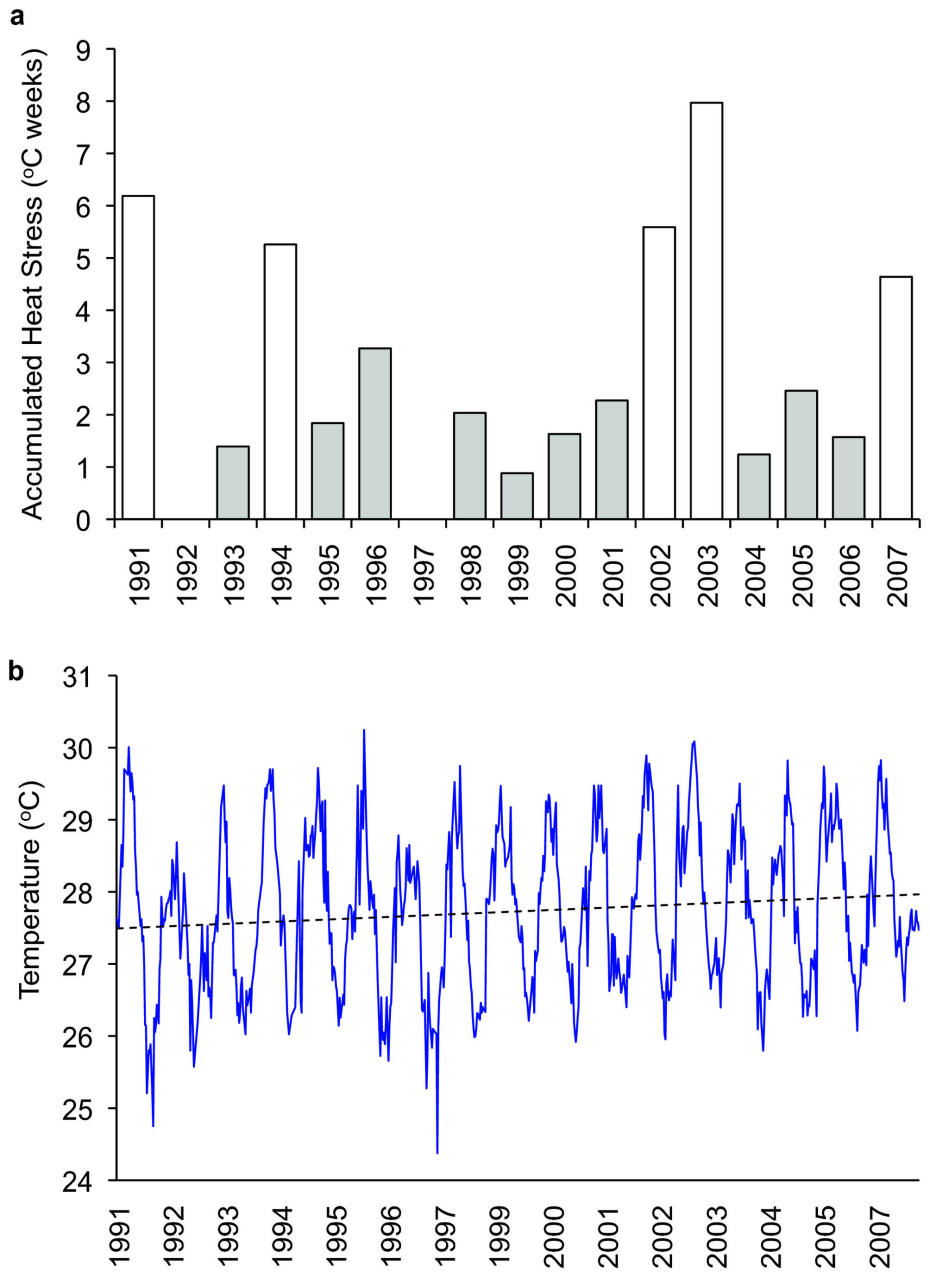
Annual thermal conditions in Moorea from 1991 to 2007. Data were derived from the NOAA Pathfinder v 5.0 dataset. a) Annual accumulated heating stress (in °C-weeks) was calculated by summing positive anomalies above the maximum monthly mean of 29.0 °C. Years where bleaching was observed are shown in white. b) The long-term trend in daily temperature reveals an increase during the study period at a rate of 0.16 °C per decade.

From February 2007, coral assemblages on the north coast of Moorea were subjected to a prolonged period (11 of 12 consecutive weeks) of water temperatures in excess of 29.0°C. These conditions caused accumulated heat stress (in °C-weeks) of 4.63; less than the 7.96 experienced in 2002, but comparable with the 5.25 and 6.18 experienced in 1994 and 1991, respectively (Figure 1a). In 2007, 32.8% (n = 2180) of colonies were bleached. There were also low levels of recent coral mortality. However, this mortality cannot be unequivocally attributed to temperature stress and bleaching because there were also moderate densities of the coral feeding starfish *Acanthasterplanci* , in areas where bleaching was observed [29].

**Figure 2:**
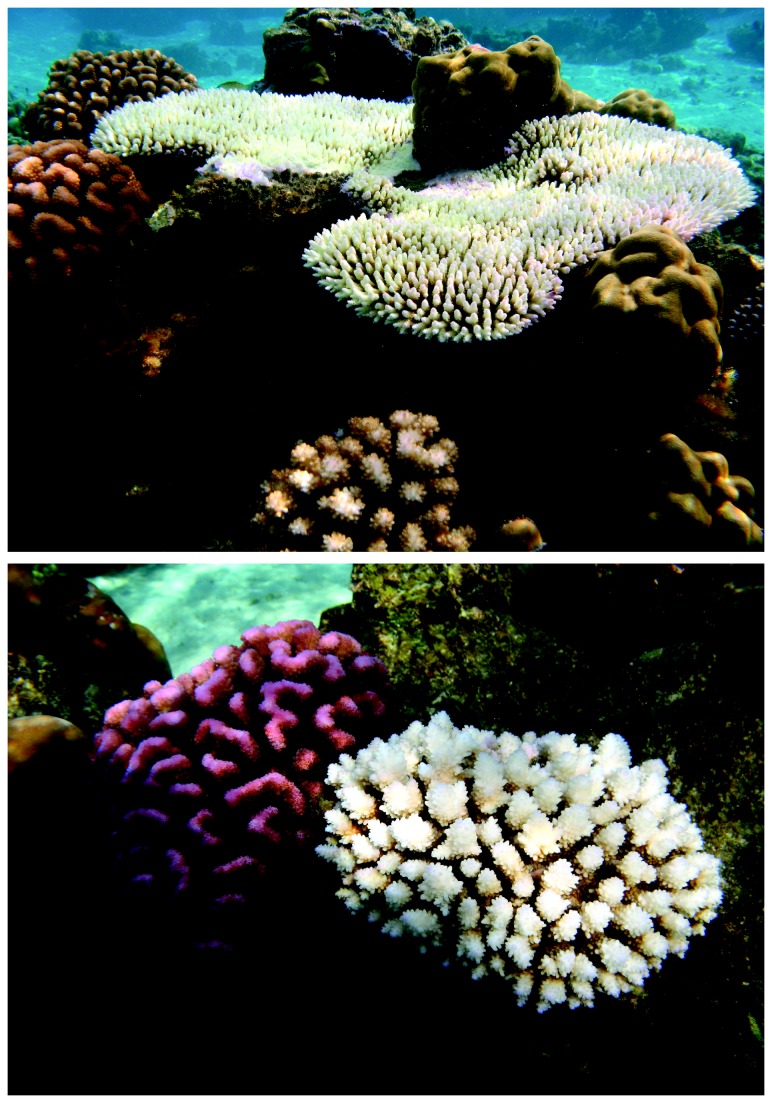
Taxonomic differences in bleaching susceptibility in Moorea, French Polynesia. Taken in late summer 2007, these photographs show bleached *Acropora* adjacent to colonies of *Pocillopora* and *Porites*, which are seemingly unaffected. *Pocillopora* are generally considered to be amongst the most susceptible genera to climate-induced coral bleaching (e.g., McClanahan et al. 2004), but *Pocillopora* corals exhibit unusually high resistance to high temperatures at Moorea.

**Figure 3:**
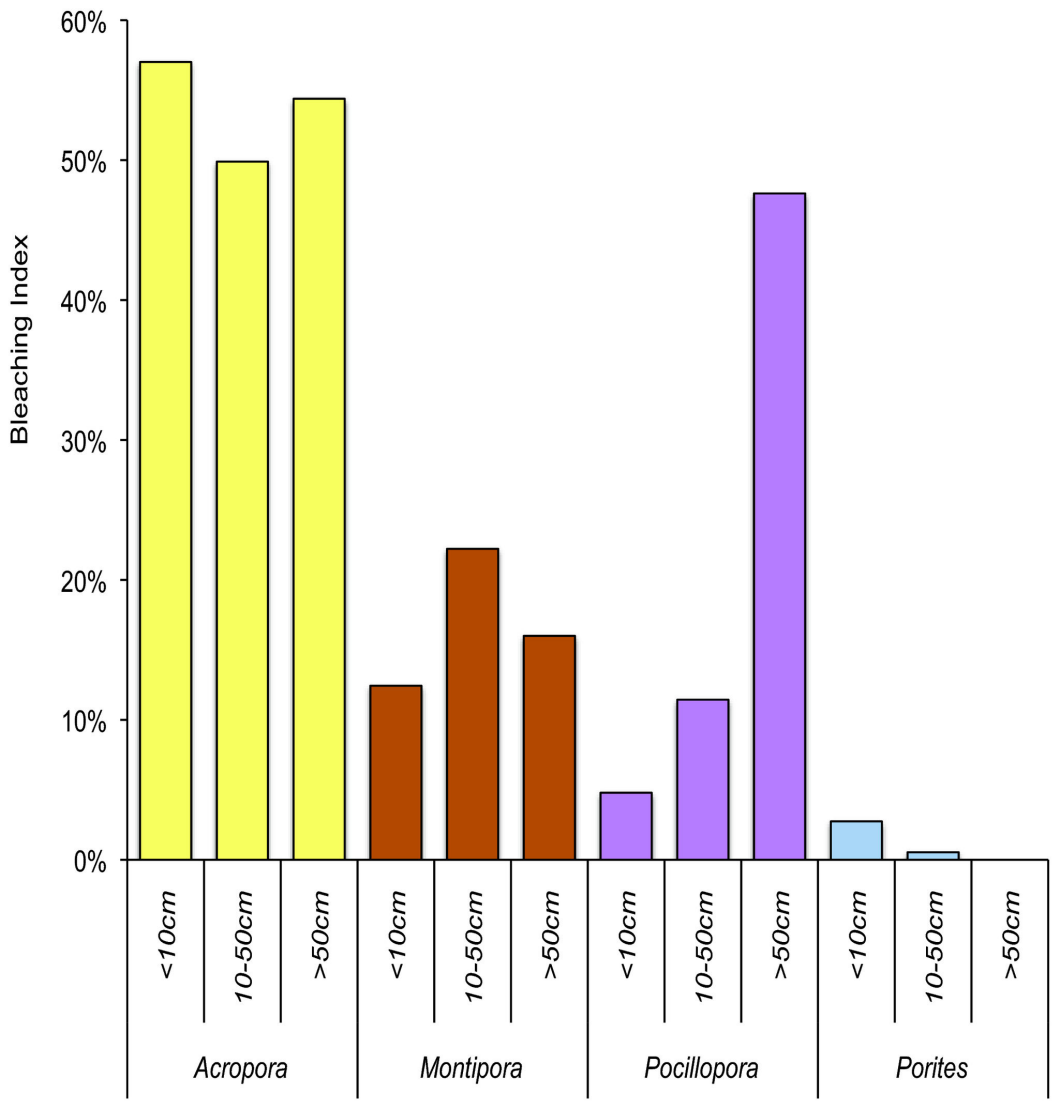
Size-specific bleaching susceptibility for four key genera (*Acropora*, *Montipora*, *Pocillopora*, and *Porites*) in 2007. Bleaching susceptibility was calculated using a bleaching index (BI) that weights the proportion of colonies that bleached by the severity of bleaching, following Guest et al. (2012).

Taxonomic differences in bleaching susceptibility were very pronounced (Figure 2): 64.9% (n = 548) of colonies of *Acropora* were bleached, whilst only 2.44% (n = 286) of *Porites* colonies exhibited any evidence of bleaching. Moreover, most *Acropora* were completely (100%) bleached, whereas most bleached colonies of *Pocillopora* and *Porites* were only partially bleached. Bleaching susceptibility was greatest for *Acropora* (*BI* = 51.87), then *Montipora* (*BI* = 19.30), *Pocillopora* (*BI* = 13.81), and *Porites* (BI = 0.84). Analysis of the bleaching and mortality response (BMI) across these four major coral genera revealed significant differences in size-specific bleaching susceptibility (Friedman test = 40.38, df = 3, p < 0.01). For *Acropora* and *Montipora*, bleaching susceptibility was fairly consistent across small, medium and large colonies (Figure 3). For *Pocillopora* and *Porites* however, there was a marked effect of colony size on bleaching susceptibility. In *Pocillopora*, large colonies (>50 cm diameter) had a much higher susceptibility (*BI* = 47.62) compared with smaller colonies (Figure 3). For *Porites*, bleaching was restricted to small and medium sized corals (<50 cm diameter), with no bleaching recorded amongst colonies >50cm in diameter (Figure 3).

### History of bleaching

**Figure 4:**
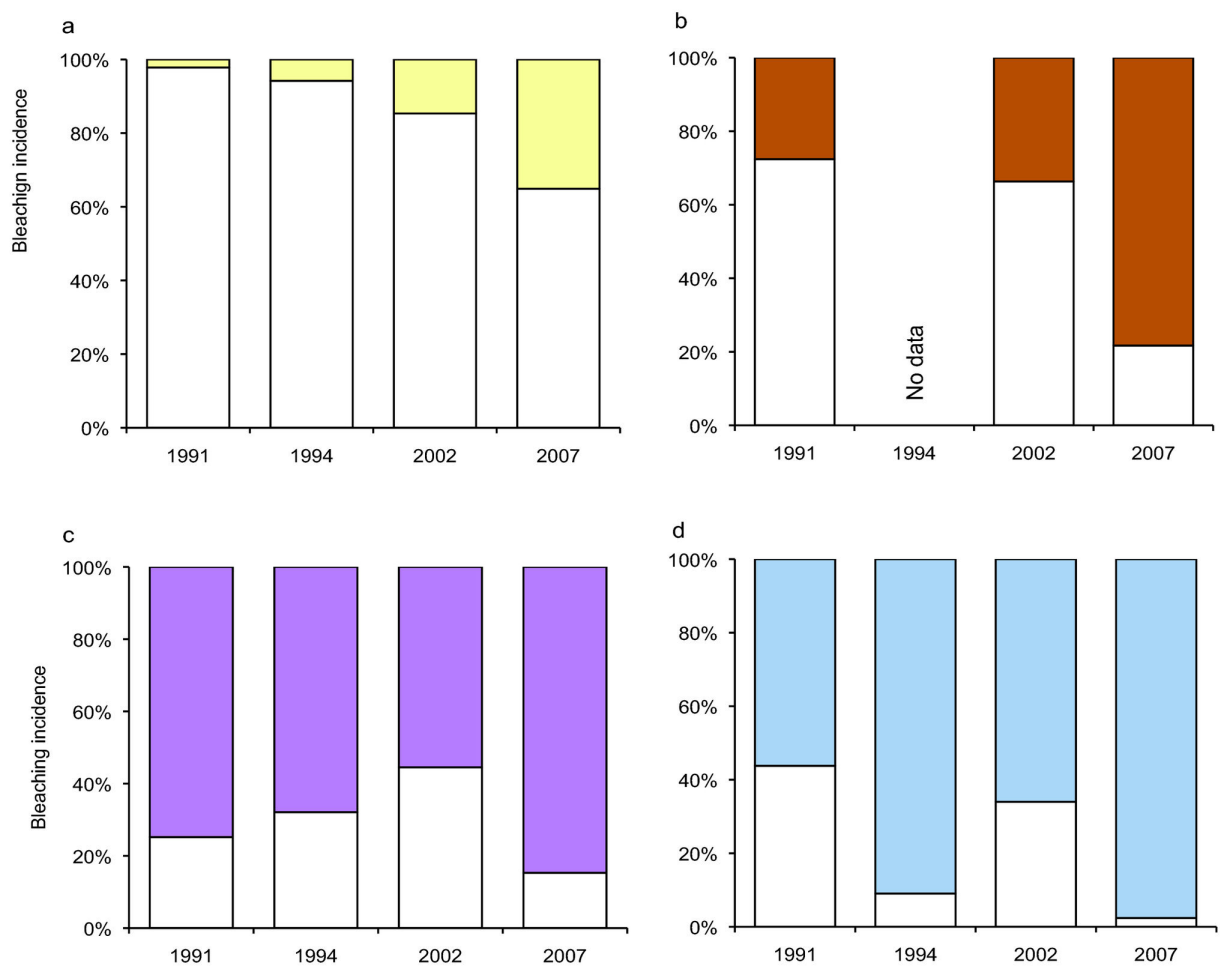
Proportional bleaching in a) *Acropora*, b) *Montipora*, c) *Pocillopora*, and d) *Porites* during well-documented bleaching events in Moorea, French Polynesia. Graphs distinguish the proportion of colonies for each genus that had any evidence of bleaching (in white) from those that did not bleach (coloured) in 1991, 1994, 2002 and 2007. All surveys were conducted on the outer reef slope (6-18m depth) along the northern coast of Moorea.

Since 1979, coral reefs on the north coast of Moorea have been subject to several major disturbances, including seven bleaching events, two cyclones and two major outbreaks of *Acanthasterplanci* [26]. The best documented bleaching events occurred in 1991 [30], 1994 [31], 2002 [27] and 2007 [29]. On each of these occasions, the proportion of colonies that bleached tended to be recorded for each of the major coral genera (e.g., *Acropora*, *Montipora*, *Pocillopora* and *Porites*). Analysis of the bleaching incidence for these four major coral genera revealed significant change in the relative susceptibility of taxa among years in which bleaching was documented (Friedman test = 39.67, df = 3, p < 0.01). Importantly, the proportion of colonies that bleached during each bleaching episode was very different (Figure 4). The proportion of *Acropora* (Figure 4a) and *Montipora* colonies (Figure 4b) that bleached in 2002 was lower than in previous bleaching events and lower again in 2007. Indeed, bleaching susceptibility in these genera appears to be decreasing through time. There also appears a similar trend for *Porites* (Figure 4c), but not for *Pocillopora* (Figure 4d).

### History of environmental parameters in Moorea

Higher maximum temperatures, and/or prolonged exposure to unusually high temperatures typically results in much higher incidence of bleaching and mortality [7]. Accordingly, bleaching has been observed at Moorea in years (1991, 1994, 2002, 2003, and 2007) when accumulated heat stress is highest and at least above 4 (Figure 1a). The accumulated heat stress in these five years was significantly higher (Student’s t = 61.65, df = 15, p = 1.08e-6) than for all other years when no bleaching was recorded. Average annual temperatures have increased gradually (0.16 °C per decade) since 1991 (Figure 1b). The heat stress experienced in 2003 and 2007 is comparable with or higher than in 1991 and 1994 but the severity of mass bleaching (the proportion of colonies bleached) has declined from 1991 to 2007.

**Figure 5:**
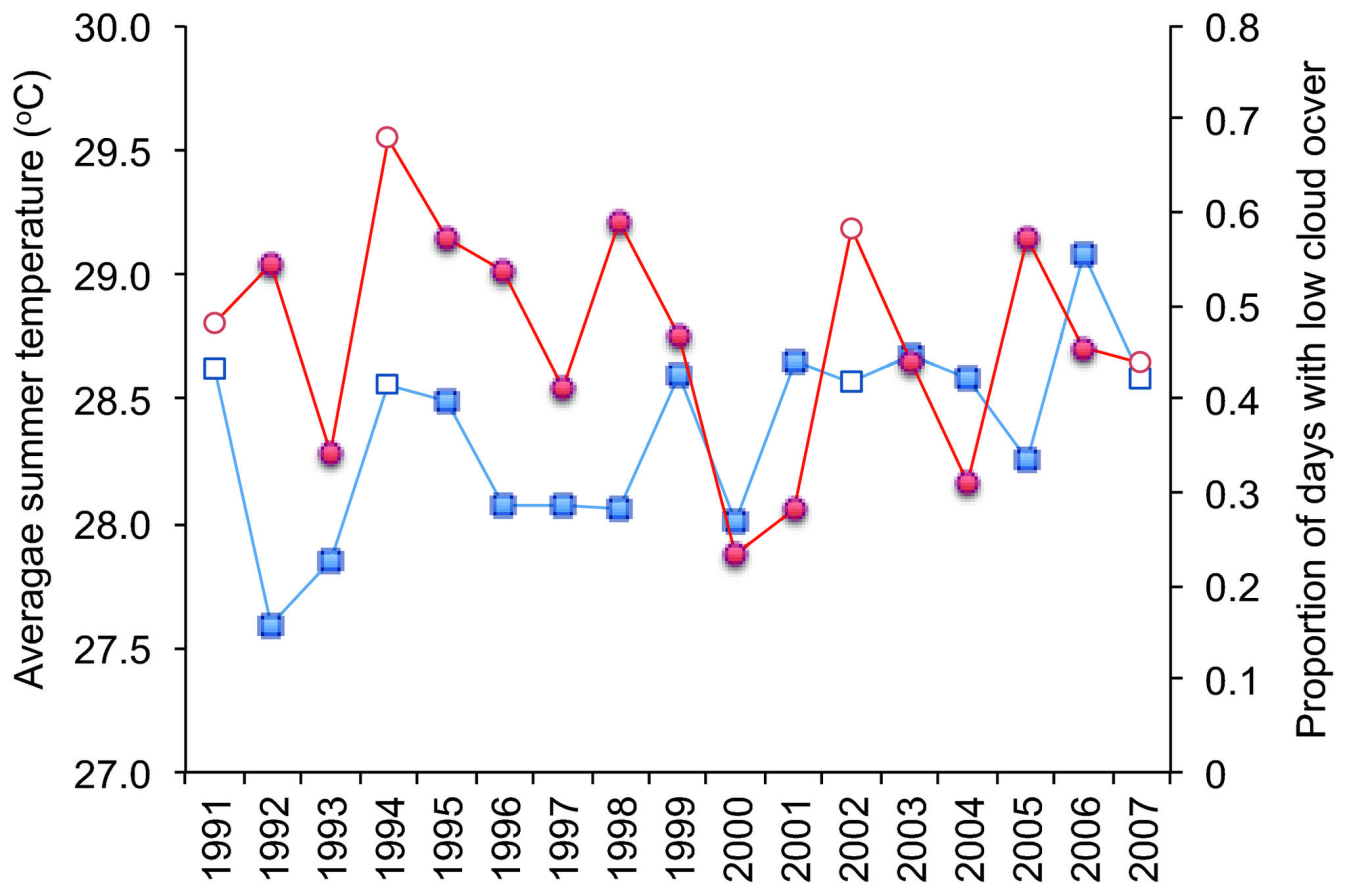
Summer temperature and cloud conditions at Tiahura Reef, Moorea, from 1991 to 2007. Average sea surface temperature (red circles) and the proportion of early-afternoon summer cloud cover observations less than the summer median (blue squares). Open symbols represent the four bleaching events considered in 1991, 1994, 2002 and, 2007.

Temporal declines in bleaching incidence reported for *Acropora* and *Pocillopora* during the four well-documented bleaching events in Moorea (Figure 4) are not clearly linked to successive declines in the local levels of average temperature or higher cloud cover (Figure 5). There was also no significant differences in annual summer temperature for the five years when bleaching was observed and the remaining non-bleaching years (Student’s t = 2.887, df = 15, p = 0.11). The synergistic effects of high light levels and high temperature on coral bleaching [32] suggests that cloud incidence could influence the extent and severity of observed bleaching. However, the percentage of summer cloud cover observations less than the median value was similar between 1991 and 2007; and the difference between the five analysed bleaching years (including 2003) and the remaining (non-bleaching) years was not significant (Student’s t = 1.608, df = 15, p = 0.224).

## Discussion

Bleaching incidence recorded in each of four major coral genera (*Acropora*, *Montipora*, *Pocillopora* and *Porites*) in 2007 was much lower compared with previously documented mass bleaching at Moorea. The overall proportion of corals bleached in 2007 (25% of colonies across all species) was approximately half that recorded in 1991 (51%). For *Acropora*, which is typically the genus most susceptible to coral bleaching [23], there was a 30% decline in bleaching incidence from 1991 to 2007 and a relatively consistent decline in the proportion of colonies that bleached over the four well-documented bleaching events. There are a number of environmental factors that may moderate bleaching responses during periods of high temperatures, particularly high cloud cover [32–34]. We failed to find any systematic trend in local environmental conditions (e.g., temperature or cloud cover) that might account for the sustained declines in bleaching susceptibility observed for highly susceptible genera, *Acropora* and *Montipora*. It is possible therefore, that recurrent bleaching has resulted in increased resistance to bleaching especially among the most susceptible genera (e.g., *Acropora*), as has been reported elsewhere [24,25,35].

Bleaching susceptibility varied greatly within and among coral genera on reefs in Moorea, with the potential to alter both population and community structure [33]. With increasing environmental stress, it is expected that coral populations will have faster turnover and become increasingly dominated by small corals [34]. Changes in population structure may be further reinforced by size-based differences in bleaching susceptibility [33,35], as was evident among *Pocillopora* corals at Moorea (Figure 3). However, size-selectivity in bleaching susceptibility is not always apparent [36] and was not observed for *Acropora*. It is suggested that smaller and flatter corals have a greater mass-transfer capacity, corresponding with greater resistance to bleaching [37]. However, this does not effectively account for observed inter- or intra-generic differences in bleaching susceptibility (see also 38). Many such generalities in patterns of bleaching susceptibility lack empirical support [23], emphasizing the need for much greater research on individual susceptibilities of different corals.

Variation in bleaching susceptibility may also be attributable to differences in the predominant type of zooxanthellae hosted by corals [25,36]. In the eastern Pacific, for example, increasing thermal tolerance of *Pocillopora* was linked to increased prevalence of colonies that host a thermally tolerant clade D symbiont [25]. Similarly, *Pocillopora* in French Polynesia host a diversity of symbionts, including clade D [39], which may explain their low level of bleaching susceptibility compared with many other geographic locations [40]. For other coral genera, which may be incapable of switching symbionts [41], prior exposure to environmental extremes may have stimulated photo-protective mechanisms (e.g., increased concentrations of certain pigments) that reduce bleaching susceptibility [42,43].

Distinguishing between individual acclimation versus selective mortality and directional changes in the structure of coral assemblages requires detailed information on the long-term bleaching susceptibility and subsequent fate of individually tagged corals [23]. However, recent changes in the community structure of coral assemblages in Moorea [26,29,44,45] may reflect selective removal of susceptible phenotypes. Notably, Pichon [46] recorded 39 species of *Acropora* from French Polynesia, during surveys conducted prior to 1981, when *Acropora* was the dominant coral genera. In comparison, biodiversity assessments conducted in the late 1990’s recorded only 22 *Acropora* species in French Polynesia [47]. Contrasting estimates of species richness between these two studies are probably due partly to differences in methodologies, including sampling intensity and range of habitats actually sampled. However, local abundance of *Acropora* has declined substantially (>80%) since 1979 [26], and it is likely that this has reduced coral diversity. Rapid adaptation through selective removal of susceptible genotypes is also likely to be most pronounced in populations or taxa that typically experience high levels of bleaching-related mortality, like *Acropora*.

Coral reef ecosystems are widely regarded to be among the most threatened ecosystems, due to increases in ocean temperatures and extreme temperature sensitivities of most reef-building corals [8]. However, observed declines in bleaching susceptibility among reef-building corals suggest that there is some capacity for adaptation, which will delay devastating effects of global climate change. The critical question is how far can the adaptive capacity of scleractinian corals extend? Inherent limits to the rate or extent of acclimation and adaptation may simply delay local and global extinctions of coral species subject to ever increasing ocean temperatures [36]. Further, bleaching events coincident with periods of anomalously high temperatures are one of many selective forces on reefs. Gradual acclimation and adaptation to increased temperatures by coral assemblages in Moorea and elsewhere can easily be undone by other natural and anthropogenic stressors and disturbances. Outbreaks of crown-of-thorns starfish, *Acanthasterplanci* , for example, may have altogether different selective forcing on coral population and communities.

The most pronounced declines in coral cover observed in Moorea since 1979 have both been associated with outbreaks of *A* *. planci* [26]. Most recently, outbreaks of *A* *. planci* occurred in 2006, reducing total coral cover by >50% and further reducing coral diversity [29]. Moreover, outbreaks of *A* *. planci* have had a disproportionate effects on *Acropora* corals, leading to marked changes in the coral communities since 1979 [29]. Mortality caused by these disturbances is likely to have eliminated some or even all of the increased thermal tolerance gained between successive bleaching events. The capacity for scleractinian corals to adjust to, and cope with, ongoing increases in ocean temperatures may be appreciable [16,24,48]. In order to maximize adaptive capacity to climate change it will continue to be important to minimize the diversity, frequency and severity of other anthropogenic disturbances that also effect coral reef organisms.

## Materials and Methods

### Ethics statement

This study was authorised by the French Polynesia provincial government, permitted through Richard B. Gump South Pacific Biological Research Station in Moorea. Only visual censuses of fish and benthic communities were conducted during this study; no fauna or flora were collected or manipulated.

### Study sites

This study was conducted on the northern coast of Moorea (17° 30' S, 149° 50' W), Society Islands, French Polynesia. Sampling was conducted at two locations, Vaipahu and Tiahura, separated by approximately 2 kilometres on the north coast of Moorea (Pratchett et al. 2011). Sampling was conducted in six distinct reef zones: (1) the inner reef flat (1–2 m depth); (2) the outer reef flat (1–3 m depth), (3) the reef crest (3–5 m depth), (4) the shallow reef slope (7–9 m depth), (5) the mid-slope (10–12 m depth), and (6) the deep slope (18–20 m depth). Corals were sampled using five replicate (50 × 4-m) belt transects, classifying all corals to one of four different bleaching categories; (1) no bleaching, (2) moderately (<50%) bleached, (3) mostly (>50%) bleached, and (4) completely (100%) bleached, based on the proportion of the tissue area that was conspicuously pale or white, but not dead. All corals were also identified to genera and categorized to one of three different size classes; (1) <10cm maximum diameter, (2) 10-50cm maximum diameter, and (3) >50 cm maximum diameter. Bleaching susceptibility was calculated using the bleaching index (BI) that weights the proportion of colonies that bleached by the severity of bleaching [24]. A non-parametric Friedman test was carried out to compare bleaching and mortality index (BMI) values among size classes for each of the four major coral genera (*Acropora*, *Montipora*, *Pocillopora* and *Porites*).

### History of bleaching

Taxonomic differences in bleaching susceptibility, as well as absolute rates of coral bleaching, recorded in 2007 were compared with well-documented bleaching events over the past 2 decades. Changes in coral cover and composition (mostly to genus) have been reported in Moorea since the 1970s, focusing on Tiahura reef (and to a lesser extent at Vaipahu) on the north-west corner of Moorea [26]. During this period, coral assemblages have been subject to many acute (pulse) disturbances, which have caused major changes in the community structure. Most notably, multispecific coral bleaching has been reported every 3-4 years since 1983 (1984, 1987, 1991, 1994, 2002, 2003, and 2007), corresponding with periods when sea surface temperature increased above 29.0 °C [27,31,44]. However, the best documented instances of bleaching, including estimates of proportional bleaching in each of the major coral genera, occurred in 1991 [30], 1994 [31] 2002 [27] and 2007 [29]. For each of these studies, we used data on the proportion of colonies within each of four major genera that had exhibited bleaching. All four studies report on proportional bleaching for major coral genera on the outer reef slope. Of the available data we have restricted attention to sites (i.e., Tiahura and Viapahu) on the north coast, with sampling conducted at depths between 6 and 18m. A non-parametric Friedman test was carried out to compare bleaching susceptibility among four major coral genera (*Acropora*, *Montipora*, *Pocillopora* and *Porites*) across years (1991, 1994, 2002, and 2007).

Sea surface temperature data for the summer periods (Feb–Apr) between 1991 and 2007 were acquired from the NOAA Pathfinder v 5.0 dataset and weekly night-only composites were gapfilled following Heron et al. [49]. The temperature time-series for the 4×4 km pixel closest to the centre of the two study sites was extracted to determine the summer average temperature. The threshold for thermal stress was defined as the maximum monthly mean (MMM = 29.0 °C [50]) of the dataset and accumulated heat stress (in °C-weeks) was calculated for all temperatures exceeding the MMM value. Accumulated heat stress is similar to a ‘degree heating week’ (which accumulates only when temperature is MMM +1 °C or above [50]) in that one week at 1 °C above the MMM results in 1 °C-week of accumulated heat stress. Comparison of annual summer temperature and accumulated heat stress during bleaching years and from non-bleaching years was undertaken using Student’s t-test.

Cloud data for the summer periods (Feb–Apr) between 1991 and 2007 were acquired from the International Satellite Cloud Climatology Project (ISCCP; isccp.giss.nasa.gov [51]) for the ~250×250 km pixel that included the study sites. Cloud cover values in the early-afternoon (local time 12: 00 and 15: 00) were analysed following Mumby [32]. The median of these summer cloud cover observations during the 17-year study period was determined as 27.7%. To evaluate the impact of light exposure on the observed levels of bleaching, the proportion of early-afternoon summer cloud cover observations less than 25% (i.e., higher than median insolation) was determined for Feb–Apr of each year (Figure 5). Comparison of cloud cover proportion during bleaching years and from non-bleaching years was undertaken using Student’s t-test.
